# Correction: Reinvestigation of Passerini and Ugi scaffolds as multistep apoptotic inducers *via* dual modulation of caspase 3/7 and P53-MDM2 signaling for halting breast cancer

**DOI:** 10.1039/d4ra90143f

**Published:** 2024-12-11

**Authors:** Mohammed Salah Ayoup, Yasmin Wahby, Hamida Abdel-Hamid, Marwa M. Abu-Serie, Sherif Ramadan, Assem Barakat, Mohamed Teleb, Magda M. F. Ismail

**Affiliations:** a Chemistry Department, Faculty of Science, Alexandria University P. O. Box 426 Alexandria 21321 Egypt mohammedsalahayoup@gmail.com Mohamed.salah@alexu.edu.eg; b Medical Biotechnology Department, Genetic Engineering and Biotechnology Research Institute, City of Scientific Research and Technological Applications (SRTA-City) Egypt; c Chemistry Department, Michigan State University East Lansing MI 48824 USA; d Department of Chemistry, Benha University Benha Egypt; e Department of Chemistry, College of Science, King Saud University P. O. Box 2455 Riyadh 11451 Saudi Arabia ambarakat@ksu.edu.sa; f Department of Pharmaceutical Chemistry, Faculty of Pharmacy, Alexandria University Alexandria 21521 Egypt; g Department of Pharmaceutical Medicinal Chemistry, Faculty of Pharmacy (Girls), Al-Azhar University Cairo 11754 Egypt

## Abstract

Correction for ‘Reinvestigation of Passerini and Ugi scaffolds as multistep apoptotic inducers *via* dual modulation of caspase 3/7 and P53-MDM2 signaling for halting breast cancer’ by Mohammed Salah Ayoup *et al.*, *RSC Adv.*, 2023, **13**, 27722–27737, https://doi.org/10.1039/d3ra04029a.

The authors regret an error in Fig. 2 where two of the panels contain partial overlap. The panels for 8-treated MDA-MB 231 and 12-treated MCF-7 cells contain overlap as it was found that two images with different orientations or poses of Y50MD (original code of 12-treated MDA-MB 231 cells) were mistakenly renamed in the final folder.

In addition, while reviewing raw images, it was noticed that other raw images (4-treated breast cancer cells and 8-treated MCF7 cells) were not correctly placed in their corresponding panels in the final Fig. 2.

The figure should have been:

**Fig. 1:**
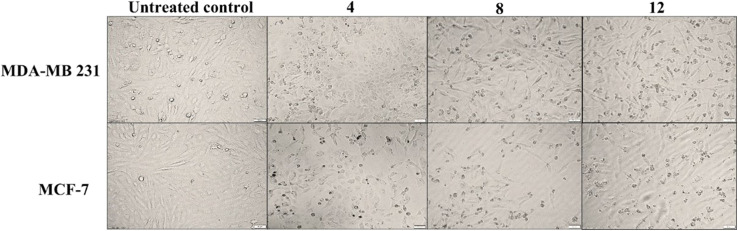
Morphological changes of breast cancer cells after 72 h treatment with the promising hits.

The Royal Society of Chemistry apologises for these errors and any consequent inconvenience to authors and readers.

